# The mental health crises of the families of COVID-19 victims: a qualitative study

**DOI:** 10.1186/s12875-021-01442-8

**Published:** 2021-05-15

**Authors:** Fateme Mohammadi, Khodayar Oshvandi, Farshid Shamsaei, Fateme Cheraghi, Masoud Khodaveisi, Mostafa Bijani

**Affiliations:** 1 Chronic Diseases(Home Care) Research Center and Autism Spectrum Disorders Research Center, School of Nursing and Midwifery, Department of Nursing, Hamadan University of Medical Sciences, Hamadan, Iran; 2 Mother and Child Care Research Center, School of Nursing and Midwifery, Department of Nursing, Hamadan University of Medical Sciences, Hamadan, Iran; 3 Behavioral Disorders and Substance Abuse Research Center, Institute of Mental Health and Addiction, School of Nursing and Midwifery, Department of Nursing, Hamadan University of Medical Sciences, Hamadan, Iran; 4 Chronic Diseases (Homecare) Research Center, School of Nursing and Midwifery, Department of Nursing, Hamadan University of Medical Sciences, Hamadan, Iran; 5 Clinical Research Development Unit, Valiasr Hospital, Fasa University of Medical Sciences, Fasa, Iran

**Keywords:** Mental health crisis, Victims, Family, COVID-19, Qualitative research

## Abstract

**Background:**

The bereaved families of COVID-19 victims are among the most vulnerable social groups in the COVID-19 pandemic. This highly infectious and contagious disease has afflicted these families with numerous psychological crises which have not been studied much yet. The present study is an attempt at investigating the psychological challenges and issues which the families of COVID-19 victims are faced with. The present study aims to identify the Mental Health crises which the families of COVID-19 deceased victims are going through.

**Methods:**

A qualitative research, the present study uses a conventional content analysis design. The participants were 16 members of the families of COVID-19 victims selected from medical centers in Iran from February to May 2020 via purposeful sampling. Sampling continued to the point of data saturation Data were collected via semi-structured individual interviews conducted online. The collected data were analyzed according to the conventional qualitative content analysis approach.

**Results:**

Analyses of the data yielded two main themes and seven categories. Emotional shock included (feelings of guilt and rumination, bitter farewell, strange burial and concern about unreligious burial), and fear of the future included (instability in the family, lack of job security and difficult financial conditions, Stigmatization and complications in social interactions).

**Conclusion:**

The families of COVID-19 deceased victims are affected by various psychological crises which have exposed them to a deep sense of loss and emotional shock. Therefore, there is an urgent need for a cultural context which recognizes and supports all the various aspects of the mental health of these families.

## Background

COVID-19 has been the most horrifying emerging disease in recent decades [1, 2], spreading extreme fear across the whole world, Because in previous decades there was no pandemic that infects many people around the world [3]. Emerging diseases are diseases which appear for the first time in a certain area or the world, have high severity, and quickly infect a large population [4, 5]. According to World Health Organization in recent years, more than 30 emerging infectious diseases have appeared in different parts of the world and each of them has been of significance based on its type, spread, and seriousness [3, 6]. Since there is not a specific treatment for most emerging diseases, they take on epidemic proportions and spread across wide geographic areas, increasing the number of infections and deaths and, consequently, imposing considerable expenses on healthcare systems [7]. A case in point is COVID-19 which started from China in 2019 and continued to spread widely in over 200 countries, including Iran [8].

The initial symptoms of COVID-19 are similar to those of the flu, but the infection gradually spreads and affects the cardiopulmonary and renal systems. The infected often have signs of dyspnea, tachypnea, and respiratory failure, but, so far, no treatments or vaccines have been developed for COVID-19 and the patients can only be given supportive care [9, 10]. The little-known nature of COVID-19, constant changes in the genetic structure of the virus, occurrence of new symptoms in the infected, and, most importantly, lack of a definite treatment or specific vaccine for the disease have led to the infection of more than 93 million people worldwide in the past three months, 2000,000 of whom in the world and 58,000 in Iran have lost their lives to this emerging disease [3, 10].

The high rate of infection and fatality of COVID-19 in the world has exposed humans to various psychological crises with very adverse effects on their lives, social activities, and, consequently psychological security [2, 11–14]. The families of the victims, living or dead are among the social groups especially affected by psychological stress and tension [12, 13, 15]. The death of a family member usually causes emotional shock and trauma to the other members of the family who need to receive wide emotional support from relatives and even the society to adapt to their sense of loss [16, 17]. However, the sudden death of a family member to COVID-19, especially when the victim is young and does not have any underlying medical conditions, can subject families to extra shock and distress [11]. Because COVID 2019 is the most threatening pandemic in recent decades, which has caused indescribable fear among the people due the rapid transfer, and high lethality. So that peoples stay away from patients with COVID2019 and even their families [11, 13]. Obviously, the created fear affects the social interactions, work situation, family life and psychological conditions of the family members of these deceased. they experience many psychological crises and in the process of adaptation face many problems and challenges that these challenges are more than one psychological shock from the death of their family members, and their mental health is severely threatened [2, 11–13]. Furthermore, the majority (about 60%) of the deceased in Iran has been male and head of the family, responsible for the financial support of their families, decision making, and planning the future of their children [12, 13, 18, 19]. Obviously, the sudden loss of the head of one's family to COVID-19 exposes all the members of the family to psychological crises which adversely affect their adaptation, personal lives, and future. Identification of these crises and challenges can help relatives and caregivers take effective measures toward providing the families of the victims with better care and support. Accordingly, the present study aims to identify the psychological crises which the families of COVID-19 victims are faced with. Hopefully, the findings of the study can help healthcare mangers and caregivers facilitate the adaptation of the families of the deceased victims by providing them with proper mental health care.

Therefore, the present study, that is the first qualitative study on the families of COVID 2019 victims, was done with aim to identify the mental health crises of the families of deceased victims of this disease.

## Methods

The present study uses a conventional qualitative approach. Conventional content analysis is generally used with a study design whose aim is to describe a phenomenon, in this case the emotional reactions of patients and their family [20]. This type of design is usually appropriate when existing theory or research literature on a phenomenon is limited. Researchers avoid using preconceived categories, instead allowing the categories and names for categories to flow from the data. Researchers immerse themselves in the data to allow new insights to emerge [20, 21]. As the mental health crises of the families of COVID-19 victims have never been studied before, the researchers applied conventional content analysis. The study subjects were 16 members of families which had lost a member to COVID-19, selected via purposeful sampling. Purposeful sampling is widely used in qualitative research for the identification and selection of information-rich cases related to the phenomenon of interest. Information-rich cases are those from which one can gather important information about issues of research. Studying information-rich cases yields insights and in-depth understanding rather than empirical generalizations [22].

The subjects consisted of 6 wives, 3 husbands, 5 children, and 2 mothers who represented a wide range in terms of relationship, gender, financial status, education, etc. Sampling lasted from February to May 2020 until rich data were collected. The inclusion criteria were: being Iranian, speaking Farsi, being able to provide adequate rich information, and having had their deceased family member hospitalized in a medical center designated for COVID-19 patients from the time of their diagnosis until death. Data were collected from 16 semi-structured individual interviews which were conducted via video call in what’s App at times which suited the participants. Each interview started with a few general questions, including: "How did the infection of your deceased family member progress?", "What were your feelings from the diagnosis to the death of your family member?", and "How has this incident (loss) affected your life?", Next, based on the participants' answers, follow-up questions were asked to add to the clarity of the information: "Will you explain further?", "What do you mean by that?", and "Will you give an example?", The interviews were managed so as to gather information related to the main objective of the study. Each interview lasted from approximately 38 to 52 min. Immediately after each interview, one of the researchers (correspond author) listened to it several times and transcribed it, then coded meaning units and extracted subcategories and categories from the interview. Thus, data were analyzed as soon as they were collected and the next interview was planned based on the results of its predecessors. Actually, If some of the participants 'statements were vague or contradictory to each other, researcher in the next interview, vague and contradictory statements that extracted from the analysis of previous interviews, asked of new participant and wanted to explain his / her opinion or give an example. The collected data were analyzed according to the qualitative content analysis approach: considering the explicit and implicit content of the units of meaning, key points in the manuscripts were extracted as open codes. Then, based on their similarities and differences, the codes were categorized and the process of abstraction was kept up until a theme was extracted [22]. The interviews continued until data saturation was reached and no new categories emerged. To improve the trustworthiness of the results, the researchers applied Lincoln and Guba's criteria [23]. Thus, the dependability and credibility of the results were increased by immersion and prolonged engagement, member check, ad peer check.

## Ethical considerations

The institutional review board of the medical universities located in the west of Iran has approved the design of the present study (approval number: 1399.256). At the beginning of each interview, the researcher introduced herself, described the goals of the study, and assured the subjects that all information would remain confidential. The researchers also assured the subjects that they were free to withdraw from the study at any stage of the research process and that their refusal to participate or withdrawal would not have any consequence for them. Then, the participants' written informed consent was obtained.

## Results

In the present study, 16 members of the families of COVID-19 deceased victims were interviewed. The participants consisted of 9 females and 7 males (6 wives, 3 husbands, 4 sons, 1 daughter and 2 mothers). The average age of the participants was 38 years. Also, the majority of them had a high school education, and they had an income of about $ 120 in each month. Two main themes—emotional shock and fear of the future—with 9 categories were extracted from the data. Table 1 shows the themes and categories.

A. **Emotional shock**
  Approximately 14 participants mentioned that the emotional shock caused by the loss of their family members to COVID-19 was very intense and the worst psychological crisis which they had ever had to deal with. To adapt to their current situation and overcome the pain, they needed systematic psychological support from healthcare teams. The theme of emotional shock consists of four categories: feelings of guilt and rumination, bitter farewell, strange burial and concern about unreligious burial.
  ***Feelings of guilt and rumination***
    The 12 bereaved families in the present study stated that the sudden death of their family members had been a catastrophe and that they were constantly engaged in ruminating on it. They continuously reflect on the manner of infection of their deceased and keep thinking that they may have transmitted the coronavirus to their families and caused the death of their loved ones. "Spent a lot of time with my friends and did not follow those rules. I keep thinking, reviewing events, ruminating about death of my father, telling myself that I could have given the infection to him" (Participant 2).
  ***Bitter farewel***
    The 13 participants mentioned that parting from their deceased family members had been the bitterest and saddest moment in their lives. Their deaths had been so sudden that they did not have the chance to talk to their loved ones and express their love to them for the last time. They only experienced a bitter farewell. “I could not even hug and kiss my wife. The farewell was bitter and unbelievable" (Participant 8).
  ***Strange burial***
    One of the most important categories under the theme of emotional shock is mournful separation. The participants 14 stated that they never expected to have to have their loved ones buried by sanitation workers in the absence of their relatives and friends and not even have the chance to embrace them for the last time. "My mother was strangely buried…my mother's body was not delivered to us, none of our relatives and friends could be there." (Participant 8).
  ***Concern about unreligious burial***
    The 12 participants pointed out that one of the worst psychological crises which they had experienced after the death of their loved ones was their concern that they would be buried in an unorthodox and unreligious manner. They stated that the only thing that could console them a little in that situation was to know that, even if they could not bury their loved ones in the company of their relatives and friends, they would be buried in a proper religious manner. "It was said that the dead due to corona virus, were buried without bathing or shroud. Non-religious and Islamic burial was a big concern to us" (Participant 14).
B. **Fear of the future**
  The other theme extracted from the data is fear of the future. Approximately 13 bereaved families of COVID-19 victims state that fear and uncertainty about the future continuously haunts them. This theme consists of three categories: instability in the family, lack of job security and difficult financial conditions, Stigmatization and complications in social interactions.
  Instability in the familyAccording to the 12 participants, death of a family member, especially the father, has a very adverse effect on the stability of the family. If these mothers want live independently or get married again, they cannot or are not allowed to stay with their children, which inflict significant psychological tension on them.
    "After the death of my husband my father-in-law has my son custody now and if I want to move in with my parents or start a new life, he won't let me take him to live with me, my family is ruined" (Participant 4).
  Stigmatization and complications in social interactionsThe 13 participants of the present study mentioned that people are terrified of interacting with the families of COVID-19 victims and would rather stay away from them. Also being stigmatized and labeled to them after the death of their loved. This is while persistence of these behaviors will subject the bereaved families to serious psychological crises. "People are terrified of this disease and try to keep away from us, and calling me "virus spreader," "miserable woman," "poor thing" and so on. These names disturb me so much" (Participant 9).
  Lack of job security and difficult financial conditionsThe 12 participants pointed out that the death of their loved ones, especially fathers, as a result of COVID-19 has threatened the financial status of their families and that they were expecting financial difficulty in earning a livelihood and continuing their education. Also stated that fear of losing their jobs and uncertainty about employment were issues which preoccupied them and caused them considerable stress. "The costs of treatment and hospital were high…. I have very bad financial conditions. i am low-ranking employee and may be fired… I have trouble financing my family" (Participant 10).

**Table 1:** Themes and subcategories extracted from content analysis

Theme	subcategories
*Emotional shock*	• Feelings of guilt and rumination • Bitter farewell • Strange burial • Concern about unreligious burial
*Fear of the future*	• Instability in the family • Stigmatization and complications in social interactions • Lack of job security and difficult financial conditions

## Discussion

Identification of the psychological crises which the bereaved families of COVID-19 victims are exposed to can guide healthcare providers in facilitating the families' adaptation and helping them recover their emotional well-being [2, 11, 12, 14]. Thus, there is need for in-depth systematic research into the psychological issues and challenges of these families. In the present study, which is the first work of qualitative research in this field, the psychological crises of the families of COVID-19 deceased victims are classified into two themes: emotional shock and fear of the future.

The two main themes extracted from this study are similar to other studies that may be due to the fact that the sudden death of any family member, young or old, healthy or sick, causes other family members to experience emotional shock, fear and anxiety. But the categories (feelings of guilt and rumination, bitter farewell, strange burial, concern about unreligious burial, stigmatization and complications in social interactions, lack of job security), are completely specific to COVID 2019, Which distinguishes findings of this study from other studies. Therefore, the discussion was organized base on these important and different findings each themes.

Emotional shock is one of the major psychological crises which were extracted from the data. The families of COVID-19 victims were deprived of the company of relatives and friends at the funeral of their loved ones, could not hold a proper funeral for their deceased, and had to watch them buried in a desolate atmosphere, all of which facts subjected them to a major emotional shock. In the present study, this theme was found to consist of the following categories: feelings of guilt and rumination, bitter farewell, parting in desolation, concern about unreligious burial, and stigmatization. The characteristics of the coronavirus, including its high contagiousness [8], had caused some of the participants to engage in rumination: they thought that they may have transferred the infection to their family members, some of whom were older than them, and caused their death. This mental catastrophization led to rumination in the families of the deceased. Rumination is associated with anxiety, distress, and other unhealthy emotional conditions which indicate the presence of negative thoughts and feelings and can result in serious psychological harms [13, 24]. Similarly, several other studies report that the death of a family member inflicts significant psychological tension on the other members of the family and relatives [13, 24, 25]. Thus, it is necessary that healthcare providers, especially psychologists and psychiatric nurses, identify the negative thoughts of the families of the victims and take steps to protect them from rumination, catastrophizing, and emotional shock toward improving the psychological health of the society [13, 25, 26]. Although loss of a family member is a painful experience, holding a funeral in the company of relatives and friends is some consolation to the bereaved and facilitates their adaptation [27, 28]. However, the last farewell of the interviewed families of COVID-19 victims to their loved ones had been very bitter: they could not be by the side of their loved ones when they were on their deathbed, could only watch from a distance when they were being buried, and could not have the company of their friends and relatives in a proper funeral. There are many other studies which report similar results: the families of the terminally ill have to part from their loved ones in a mournful manner; however, none of those studies mentions desolation in the experiences of the bereaved [28–30]. Strange burial could have occurred during other epidemics and pandemics in the past, e.g. the Plague, cholera, etc. But no study has been found to address this issue. The participants were also concerned that their deceased would be buried in an unethical and unreligious manner. The bereaved families expected their loved ones to be buried according to Islamic rites and believed that that was the least they could do for them in those critical times. In addition, a religious burial could bring some consolation to the bereaved and help them cope with their emotional shock and distress. The participants' insistence on a proper religious burial was probably rooted in the prevalent Islamic culture of Iran: Islam dictates that the dead should be given a full-body ablution and wrapped in a shroud before they are buried with respect. In recent years, many other studies have stressed that, toward providing ethical care, caregivers should respect the preferences and religious identity of patients and their families without judging them [21, 28].

The other theme extracted from the data in the present study is fear of the future. The coronavirus knows no geographic boundaries and has afflicted the people of almost every country in the world with family, economic, and social crises; however, due to the heavy sanctions which are being imposed on Iran, Iranians are under extra pressure from these crises. The families of the infected are especially affected by this pressure [12, 19]. The participants of the present study stated that thinking about the future fills them with terror. They were worried about their family stability, job security, financial status, and social interactions with others. Instability in the family is one of the main categories under this theme. Studies conducted in other cultures show that the death of a family member, especially the father or mother, threatens stability of the family due to various reasons, including financial issues and remarriage of a parent [31, 32]. However, certain peculiarities in the culture and law of Iran make the bereaved families of COVID-19 victims, the wives in particular, especially worried about the stability of their families. The majority of the infected and the deceased in Iran are male and men are considered the lawful guardians of their children in the Iranian culture. In case of death of a child's father, his/her paternal grandfather or uncle becomes the lawful guardian and this fact puts the wives of COVID-19 victims with children in a crisis. Most of the times, if the mother decides to remarry these lawful guardians do not let the mother keep her child, which is a threat to the stability of the family [6, 33, 34]. On the other hand, though many people have faced financial problems in this crisis [19] the medical costs which were imposed on the families of COVID-19 victims and, if the victim was the head of the family, the loss of their source of income have put them under additional pressure financially. Earning a livelihood and paying for their children's education have become their biggest bugbear. Similarly, other studies report that the illness and hospitalization of one their members can strain the finances of many families, thus the need for the government or charity organizations to provide them with financial support. The fact that COVID-19 is as yet incurable and there is not a vaccine for it and the possibility that a vaccine will not be discovered for it in the near future have caused people to avoid the infected and their families, not want to have any interactions with them and even stigmatize them. On the same line, Bijani et all stated that caregivers and the whole society should treat with these patients and their family humanely and ethically and avoid all forms of stigmatization [13].

In conclusion, the bereaved families of COVID-19 victims are going through serious psychological crises as a result of the sudden loss of their loved ones, parting from them in desolation, social exclusion, and concerns over their family stability and job security. Accordingly, it is urgent that the healthcare system develop programs and protocols to protect the psychological and emotional well-being of this group.

## Limitations

One of the limitations of the present study is that, due to the high contagiousness of COVID-19 and the need for work in virtual environments at the time the study was being carried out, data were collected through personal interviews conducted via video call. Employment of other methods of data collection could have enriched the results of this work of qualitative research. It is suggested that future studies use, in addition to personal interviews, other methods of collecting qualitative data, including field notes, observation, and focus group interviews.

## Conclusion

The spread of the coronavirus across all the provinces of Iran and the increasing number of the infected and deaths have presented the healthcare system of Iran with the biggest clinical challenge in the past decade. In this crisis, one of the important tasks of the healthcare system is to maintain the psychological health of the families of COVID-19 deceased victims. Thus, identification of the factors which threaten the psychological well-being of this group is essential. The results of the present study show that the bereaved families of COVID-19 victims experience a much deeper sense of loss and emotional shock than others. Moreover, the impact of COVID-19 on the current situation in the society has filled these families with fear of the future, with adverse effects on their psychological well-being. It appears that there is need for cultural and organizational efforts toward raising the public's awareness and creating systematic health protocols to protect the psychological health of the families of COVID-19 victims. Healthcare officials and policy-makers can use the findings of the present study and provide comprehensive support (financial-social and psychological) from families of COVID-19 deceased victims, and minimize psychological distress and maintain the mental health of these families.

## Data Availability

The datasets used and/or analyzed during the current study are available from the corresponding author on reasonable request.

## Abbreviation

COVID-19: Coronavirus disease 19

## References

[1] Cao W, Fang Z, Hou G, Han M, Xu X, Dong J (2020). The psychological impact of the COVID-19 epidemic on college students in China. Psychiatry Res.

[2] Wang C, Pan R, Wan X, Tan Y, Xu L, Ho CS (2020). Immediate psychological responses and associated factors during the initial stage of the 2019 coronavirus disease (COVID-19) epidemic among the general population in China. Int J Environ Res Public Health.

[3] Coronavirus WN. Situation report–22. World Health Organization; 2019. https://www.who.int/docs/default-source/coronaviruse/situation-reports/20200211-sitrep-22-ncov.pdf.

[4] La’Toya VL, Klaphake E  (2013). Selected emerging diseases of amphibia. Vet Clin.

[5] Rodríguez-prieto V, Vicente-Rubiano M, Sánchez-MatamoroS A, Rubio-Guerri C, Melero M, Martínez-López B (2015). Systematic review of surveillance systems and methods for early detection of exotic, new and re-emerging diseases in animal populations. Epidemiol Infect.

[6] Suwantarat N, Apisarnthanarak A (2015). Risks to healthcare workers with emerging diseases: lessons from MERS-CoV, Ebola, SARS, and avian flu. Curr Opin Infect Dis.

[7] Borycki E, Cummings E, Dexheimer J, Gong Y, Kennebeck S, Kushniruk A (2015). Patient-centred coordinated care in times of emerging diseases and epidemics. Yearb Med Inform.

[8] Wu Z, McGoogan JM (2020). Characteristics of and important lessons from the coronavirus disease 2019 (COVID-19) outbreak in China: summary of a report of 72 314 cases from the Chinese Center for Disease Control and Prevention. JAMA.

[9] Ai T, Yang Z, Hou H, Zhan C, Chen C, Lv W (2020). Correlation of chest CT and RT-PCR testing in coronavirus disease 2019 (COVID-19) in China: a report of 1014 cases. Radiology.

[10] Lai C-C, Shih T-P, Ko W-C, Tang H-J, Hsueh P-R (2020). Severe acute respiratory syndrome coronavirus 2 (SARS-CoV-2) and corona virus disease-2019 (COVID-19): the epidemic and the challenges. Int J Antimicrob Agents.

[11] Mo Y, Deng L, Zhang L, Lang Q, Liao C, Wang N (2020). Work stress among Chinese nurses to support Wuhan for fighting against the COVID‐19 epidemic. J Nurs Manag.

[12] Bijani Mostafa, Gholampour Yousef, Farjam Mojtaba, Oshvandi Khodayar, Mohammadi Fateme (2020). Caregivers’ perception of the caring challenges in Coronavirus Crisis (COVID-19): A qualitative study. Med Care.

[13] Bijani Mostafa, Gholampour Yousef, Farjam Mojtaba, Oshvandi Khodayar, Mohammadi Fateme (2020). Perception of psychological safety in patients with coronavirus (COVID-19): a qualitative study. Risk Manag Healthc Policy.

[14] Nemati M, Ebrahimi B, Nemati F (2020). Assessment of Iranian nurses’ knowledge and anxiety toward COVID-19 during the current outbreak in Iran. Arch Clin Infect Dis.

[15] Xu R, Du M, Li L, Zhen Z, Wang H, Hu X (2020). CT imaging of one extended family cluster of corona virus disease 2019 (COVID-19) including adolescent patients and “silent infection”. Quant Imaging Med Surg.

[16] Ahmadian S, Khaghanizadeh M, Khaleghi E, HosseinZarghami M, Ebadi A (2019). Stressors experienced by the family members of brain-dead people during the process of organ donation: A qualitative study. Death Stud.

[17] Phongtankuel V, Burchett CO, Shalev A, Adelman RD, Prigerson HG, Czaja SJ (2019). Perceptions of a home hospice crisis: An exploratory study of family caregivers. J Palliat Med.

[18] Abdi M. Coronavirus disease 2019 (COVID-19) outbreak in Iran: Actions and problems. Infect ContrHosp Epidemiol. 2020;41(6):754-5. 10.1017/ice.2020.86 PMC7137533 32192541

[19] Takian A, Raoofi A, Kazempour-Ardebili S (2020). COVID-19 battle during the toughest sanctions against Iran. Lancet (London England).

[20] Kuckartz U. Qualitative content analysis: From Kracauer's beginnings to today's challenges. In: Forum Qualitative Sozialforschung/Forum: Qualitative Social Research. Philipps: Universität Marburg; 2019;20(3):1-19.

[21] Mohammadi F, Tabatabaei HS, Mozafari F, Gillespie M (2020). Caregivers’ perception of women’s dignity in the delivery room: A qualitative study. Nurs Ethics.

[22] Speziale HS, Streubert HJ, Carpenter HJ. Qualitative research in nursing: Advancing the humanistic imperative. Wolters Kluwer, Philadelphia: Lippincott Williams & Wilkins; 2011.

[23] Guba EG, Lincoln YS (1994). Competing paradigms in qualitative research. Handb Qual Res.

[24] Imanzadeh A (2019). Lived experiences of cancer patients from death anxiety based on Jaspers borderline situations. Iran J Psychiatr Nurs.

[25] MacLeod R, Wilson DM, Crandall J, Austin P (2019). Death anxiety among new zealanders: the predictive roles of religion, spirituality, and family connection. Omega.

[26] Russell DW, Russell CA. The stress bias in mental health reporting: Death anxiety biases mental health self-assessments amongst deployed soldiers. Psychol Serv. 2019;1(999):1-13. 10.1037/ser0000391 31512906

[27] Guerrero JG (2019). Nurses towards End-of-Life situations: Sympathy vs Empathy. Open J Nurs.

[28] Mohammadi F, Oshvandi KH, Khodaveisi M, Cheraghi F, HasanTehranid T, Khalili A (2020). Caregivers’ perception of teenagers’ dignity in end of life stages: a phenomenological study. Nurs Ethics.

[29] Vanstone M, Neville TH, Clarke FJ, Swinton M, Sadik M, Takaoka A (2019). Compassionate end-of-life care: mixed-methods multisite evaluation of the 3 wishes project. Ann Intern Med.

[30] Weisse CS, Melekis K, Hutchins B (2019). Providing end-of-life care: increased empathy and self-efficacy among student caregivers in residential homes for the dying. Am J Hospice Palliat Med.

[31] Hua P, Bugeja L, Maple M (2019). A systematic review on the relationship between childhood exposure to external cause parental death, including suicide, on subsequent suicidal behaviour. J Affect Disord.

[32] Saarela J, Rostila M (2019). Mortality after the death of a parent in adulthood: a register-based comparison of two ethno-linguistic groups. Eur J Pub Health.

[33] Keefe J, Rajnovich B (2007). To pay or not to pay: Examining underlying principles in the debate on financial support for family caregivers. Can J Aging.

[34] Mohammadi F, Rakhshan M, Molazem Z, Zareh N, Gillespie M (2019). Caregivers’ perception of dignity in teenagers with autism spectrum disorder. Nurs Ethics.

